# Psychiatry

**Published:** 1905-08-19

**Authors:** 


### Progress in Medicine and Surgery.

### PSYCHIATRY.

(*Continued from page 344.*)

**The Prevalence of Insanity in Different Races and its Biological Significance.**—A masterly exposition of the prevalence and distribution of insanity in many lands, with a special analysis of the conditions in various parts of Scotland, is given in Dr. John Macpherson's fifth Morison[4] Lecture. In the second lecture the distribution of the neuroses throughout the various races of mankind was discussed briefly, and it was then shown that no race of man was free from very extensive affection by epilepsy, hysteria, and other nervous infection. With reference to insanity in various parts of the world, travellers and chroniclers are generally silent; in many instances they have failed to record observations because the subject did not interest them, while in still other instances they simply say that they saw no insane people. A few records, by colonial and other physicians, are available which show that insanity also, like the neuroses, does exist among savage and barbarous people. Thus Dr. Felkin, of Uganda, states that he has seen in all some thirty to forty lunatics in the region of the White Nile; he also saw some maniacs in chains. He states that the type of acute insanity (mania) among the African natives is different from that in Europe. The prevailing form of mania is of a short acute kind, lasting only a day or two, during which the sufferer is driven away to the woods or voluntarily runs away, returning again in a few days apparently restored in mind. Idiocy was very common, in his experience, a fact which has been established also for a South African race, the Basutos, among whom it is highly prevalent. Suicide, adds Dr. Felkin, was also frequently committed by natives during temporary insanity. Thompson in his book "Through Masailand," states that he found insanity very common. The myths and folklore of the people were full of references to it. Those affected by insanity were driven away from the habitation of sane people, or isolated. He also found idiocy very common especially among the Masai dwarfs and albinos, the latter of whom were numerous and undoubtedly defective in mind. Captain Cook, in his "Voyages," referring to the South Sea Islanders, says:—"We met with two instances of persons of disordered mind, the one a man at Owyhee and the other a woman at Oneheeow. It appeared from the particular attention and respect paid to them that the opinion of their being inspired by the Divinity, which obtains among most of the nations of the East, is also received here" (in the Pacific).[4] Ellis in his "Polynesian Researches" says:—"Insanity prevailed to a slight decree (among natives) . . . they were supposed to be inspired or possessed by some god, . . . on this account no control was exercised, but they were treated with the highest respect. They were, however, avoided," etc. Emin Pasha, in his book "Central Africa," says:—"Insanity, and also temporary mental aberration are frequent among the natives. The latter are treated with herbal remedies which effect an immediate cure by means of sleep and sweating."[5] Wilson and Felkin ("Uganda and the Egyptian Soudan") state that "temporary madness is pretty common, and generally lasts for three or four days, but persons thus afflicted do not become very violent." Such extracts might be indefinitely multiplied. "No nation or race in the world is free from epileptic or hysterical neuroses," adds Dr. Macpherson, and if there was any difference between races it was in the extent to which they were invaded by these diseases. If such diseases as epilepsy, imbecility, hysteria, and insanity could not exist independently of the neuro- or psycho-pathic constitution, then it was the constitution itself, and not any particular manifestation of it, which we ought to endeavour to trace and understand. So far from the neuropathic constitution being considered pathological among barbarous people, its manifestations are regarded as valuable qualities and are regularly put to a marketable use. The subjects of epilepsy and hysteria supplied the ranks of the soothsayers, the magicians, the workers of miracles, and the priests. The recent studies of insanity in the Malay races by Professor Kraepelin[6] throws an interesting light on this question. A year ago he visited the State Asylum at Buitenzorg in Java. His inquiries and observations revealed to him the fact that patients of European birth born and reared in Java presented the same clinical pictures and types of mental disease as at home. (Senile dementia, however, was rare and exceptional, this fact, as in other colonies, being due to the character of the population). As to the abuse of stimulants and narcotics, the natives did not drink alcohol, and therefore cases of alcoholic insanity are not met with in the native population of the asylum. Opium-smoking and abuse of the drug is common in Java (Malays) as well as in Singapore (Chinese and Malays), yet in neither of the State asylums were there cases of insanity due to opium; of special interest also was the fact that out of 370 insane (Malay) natives there was not a single case of general paralysis, while among 50 European male inmates there were eight cases. There is no satisfactory explanation of this fact on the basis of the ordinarily accepted theory of the syphilitic causation of general paralysis, for the native of Java is not less the victim of syphilis than other similarly circumstanced people. For an explanation we must fall back upon such imperfectly understood questions as racial types and proclivities. Dementia præcox was found to be extremely frequent among the natives, and on the whole presented symptoms similar to those found in Europeans. On the other hand, mania—melancholia was rare. Hysterical and epileptic insanities, including the form known as psychical epilepsy were observed. We see then, says Dr. Macpherson, that statistical insanity in its fluctuations must depend upon the standard of intelligence of the people whose insanity is being considered, and upon their ethical and religious attitude towards disease of all kinds. The recognition of insanity as a disease by a community is very gradual, and older notions as to its supernatural origin linger for a long time even after the general acceptance of newer ideas. Like every other variation, insanity, says Dr. Macpherson, can only be held in check—" swamped out " to use a common expression—by extensive inter-crossing. New health of constitution can only be obtained if there are units to introduce new and healthy strains. There are, then, two reasons why a decreasing population favours a high lunacy rate :—(1) Because the normal age distribution of the population is greatly modified (as, *e.g.* by extensive emigration of the young and energetic males and females). (2) Because there is not sufficient compensatory introduction of new blood to check the increase of variation. This is illustrated by study of lunacy in Argyll, Caithness, and Inverness. (The marked and high lunacy ratio of Ireland points to the same conclusion.) It is necessary to point out that in dealing solely with the statistics of pauper lunacy an element of fallacy has to be reckoned with—namely, the difference in administration of the Poor-law, as well as the difference in custom in various divisions of Scotland. The fact has also to be borne in mind that when a country is being drained by emigration, the weak, the feeble-minded and insane, and the diseased are, as a rule, left behind, so that the proportion of these to the general population is increased by arithmetical progression. Exactly the reverse is seen in large urban centres which are being constantly reinforced by the addition of young adults. There remains the question of the isolation of a community, as a favouring cause of insanity and other pathogenic variations, especially marriage isolation and in-breeding. Terrible examples of this have been afforded us in the cases of the village of Eycaux with its excessive inter-marriages (in France), of the Vale of Glamorgan " the blackest spot as regards insanity in Wales, whose inhabitants were nearly all related and constantly marrying amongst each other " until it was opened up by the railway, and the studies of Drs. Ireland[7] and Brachet upon the insanities of the Royal families of Europe (Spain, Austria, and France) which from repeated inter-marriages throughout several centuries and numerous generations produced numerous classical examples of insanity and mental degeneracy.

[4] Jour. of Mental Sci., July 1905. [5] Ibid. [6] Ibid. [7] The Clot upon the Brain (London, 1896).

